# Next-generation point-of-care diagnostics for urinary tract infections: a systematic review of rapid pathogen detection methods

**DOI:** 10.3205/id000108

**Published:** 2026-09-16

**Authors:** Silvia Juste-Alvarez, José Medina-Polo

**Affiliations:** 1 Department of Urology, Hospital Universitario 12 de Octubre, Madrid, Spain

**Keywords:** point-of-care testing, urinary tract infections, antimicrobial susceptibility testing, antimicrobial resistance, phenotypic detection

## Abstract

**Background:**

Urinary tract infections are among the most prevalent bacterial infections worldwide, with *Escherichia coli* as the leading pathogen. Conventional diagnostic methods, including urine culture and antimicrobial susceptibility testing, require 48–72 hours, often delaying targeted therapy and promoting empirical antibiotic use, thereby exacerbating antimicrobial resistance.

**Objective:**

This review summarizes advances in point-of-care diagnostic technologies for rapid detection and antimicrobial susceptibility testing in localized or uncomplicated urinary tract infections.

**Methods:**

A comprehensive literature review of studies published between 2008 and 2025 was conducted using MeSH and free-text searches for “point-of-care testing”, “urinary tract infection”, “rapid diagnostics”, and related terms. Included studies described the mechanisms, performance, and clinical applicability of emerging point-of-care systems.

**Results:**

Thirty-four studies met inclusion criteria. Paper-based assays, lateral-flow tests, microfluidic devices, and hydrogel biosensors demonstrated rapid bacterial detection (from <5 min to 6 h) with variable sensitivities (10–10^6^ CFU/mL). Among commercial devices, the Sysmex PA-100 antimicrobial susceptibility testing (AST) system provided phenotypic antimicrobial susceptibility testing within 30–45 minutes and achieved over 95% specificity compared with standard microbiology, while the Lodestar DX (LAMP-based) and Bosch Vivalytic (PCR-based) systems enabled molecular identification of uropathogens and resistance genes within 40–146 minutes.

**Conclusions:**

Point-of-care diagnostic platforms substantially reduce diagnostic turnaround, improve antibiotic stewardship, and offer cost-effective alternatives to conventional culture. The Sysmex PA-100 system currently represents the most clinically validated phenotypic point-of-care tool for uncomplicated urinary infections management. Future research should focus on expanding diagnostic panels, validating clinical outcomes, and facilitating integration into primary care and antimicrobial stewardship programs.

## Background

Urinary tract infections (UTIs) are among the most prevalent bacterial infections globally, with a steadily increasing burden. According to the Global Burden of Disease Study 2021, UTI cases increased by 66.45% from 1990 to 2021, reaching approximately 4.49 billion worldwide. UTIs disproportionately affect women: up to 40% experience at least one episode during their lifetime and 10% annually, particularly between ages 16 and 35, with nearly half recurring within one year [1]. In Spain, they account for 19.9% of all community-acquired infections in acute care hospitals, imposing substantial economic costs associated with prolonged hospitalization, treatment, and mortality [2], [3], [4].

The traditional classification of UTIs as uncomplicated or complicated is being replaced by the European Association of Urology (EAU) with a system distinguishing localized (without systemic signs) from systemic infections (with fever, chills, bacteraemia, or organ involvement such as pyelonephritis or prostatitis). Risk factors including age, anatomical abnormalities, immunosuppression, catheters, and pregnancy are recognized as modifiers of disease course and prognosis. This reclassification enhances diagnostic accuracy, facilitates more rational antimicrobial use, reduces therapeutic errors, and establishes a standardized framework for clinical research and trials [5].

A major concern in UTI management remains the excessive empirical use of broad-spectrum antibiotics, particularly in primary care, where up to 90% of antibiotics in Europe are dispensed and approximately 30% are misused [6]. This practice accelerates antimicrobial resistance (AMR), a critical global health threat. Conventional diagnostic techniques such as urine culture and phenotypic antimicrobial susceptibility testing (AST) are dependable but slow, requiring 48–72 hours and frequently delaying targeted therapy. Consequently, empirical treatment is often initiated and seldom revised once results are available, promoting resistance and therapeutic failure [7].

Rapid AST is essential to optimize antimicrobial therapy. Although genotypic AST methods provide faster results through the detection of resistance genes, they are costly and limited to predefined targets. Phenotypic AST remains the gold standard, directly assessing microbial growth in the presence of antibiotics [8]. Recent developments incorporating microfluidic and nanofluidic platforms [6] have significantly shortened diagnostic timeframes, producing actionable results within hours and improving infectious disease management [9].

Among emerging solutions, point-of-care (POC) diagnostic technologies for localized or uncomplicated UTIs represent a major innovation, enabling rapid and accurate pathogen detection directly from urine samples and supporting timely, evidence-based prescribing [2]. 

In this context, we conduct a literature review with the purpose of comparing and highlighting the advantages and disadvantages of the emerging POC approaches and the relevant advances in on-site detection of pathogens’ mechanisms, suitable to be adapted to UTI diagnosis.

## Material and methods

A comprehensive literature review was conducted to evaluate current POC diagnostic methods for localized UTIs. The search was carried out using the MeSH database and the free-text fields, reviewing articles from 2008 to 2025. The following search terms were used: “recurrent urinary tract infections“, “point-of-care testing”, “urinary tract infection”, “rapid diagnostics”, and “POC UTI”. Studies will be selected in a two-step procedure. In the first step, the titles and abstracts are screened. In the second step, the full text is examined. The selected studies were analysed to describe the underlying mechanisms of action of each diagnostic approach, including enzymatic reactions, immunochromatographic assays, nucleic acid amplification techniques, and biosensor-based methods. Furthermore, commercially available POC UTI diagnostic devices were identified and systematically compared in terms of sensitivity, specificity, turnaround time, usability, and cost-effectiveness. This methodological approach allowed for a critical assessment of the advantages and limitations of each model within clinical and resource-limited settings.

## Results – summary of the evidence

The literature search identified 54 publications, of which 42 full-text articles were reviewed and 34 met the inclusion criteria. Studies excluded lacked relevance to POC diagnostics for UTIs, sufficient methodological detail, or English-language availability. Urinary pathogen detection is classified as laboratory-based (requiring sample processing) or on-site. Laboratory methods – including culture, ELISA, PCR/FISH, and MALDI-TOF – achieve high accuracy but are limited by cost, turnaround time, and dependence on culture. Conversely, POC approaches by exploiting visible colorimetric or enzymatic reactions for visual interpretation, enable rapid, low-cost, and user-friendly testing, although often at the expense of analytical precision and pathogen identification [6].

Phenotypic AST remains the diagnostic gold standard, directly observing bacterial growth in the presence of antibiotics [6]. POC diagnostic tools studies are summarized in Table 1 (Tab. 1). There are different action mechanisms: 

**Paper-based tests:** Paper-based devices provide lightweight, biocompatible platforms with detection times from <30 s to 12 h, depending on enrichment, and sensitivities of 10–10^5^ CFU/mL, achieving up to 90–100% specificity compared with PCR. Overall, paper matrices demonstrate promising sensitivity and selectivity, though performance strongly depends on the detection chemistry, assay integration, and need for enrichment. They also have limited AST capacity [10], [11], [12], [13]. 

**Lateral flow assays (LFA):** Fluid migrates laterally across membranes, triggering specific biorecognition events. Detection times ranged from minutes to hours, with sensitivities spanning 10^4^–10^6^ CFU/mL, while specificity was assured by antibody-based recognition and it depends on the detection labels and assay design. RapidBac^®^ is a direct sandwich-format LFA which reported 86% overall sensitivity (96–99% for Gram-negative bacteria) and 94% specificity in 20 minutes [14]. The Pt-Au nanoparticle LFA [15] enabled rapid visual detection within minutes, with higher sensitivity than conventional AuNP strips. The dual FITC-LFIA achieved the best numerical performance, with a detection limit of 10^4^ CFU/mL (semi-quantitative) and high specificity via monoclonal antibodies [16]. The PdNP-based LFA demonstrated a 5–10-fold sensitivity increase over AuNP systems, also with rapid readout and high specificity [17]. By contrast, the bidirectional lac-dye LFA showed the lowest sensitivity (10^6^ CFU/mL) and required 12 h pre-culture, although specificity remained adequate [18]. Overall, PdNP and FITC-based LFAs offered the greatest improvements in sensitivity, while all systems maintained reliable specificity. 

**Stack pad assays:** Using vertically aligned membranes, they achieve <5 min detection and 10^2^ CFU/mL sensitivity, 1,000× greater than ELISA; and high specificity due to multilayer selective blocking strategies [19], [20].

**Hydrogels:** Hydrogel-based biosensors, particularly chitosan hydrogels, provided rapid (5–80 min) and highly sensitive (<1 nM to 10^7^ CFU/mL) detection, highlighting trade-offs between speed, sensitivity, and accessibility [21], [22], [23], [24]. Despite promising analytical performance, clinical validation data remain limited.

These promising approaches to POC detection of pathogens have not consolidated clinical data on sensitivity, specificity, or time-to-result yet available.

Among commercial innovations, the Sysmex PA-100 AST System (Sysmex Astrego AB, Uppsala, Sweden) [25], Bosch Vivalytic UTI POCT (Bosch Healthcare Solutions GmbH, Waiblingen, Germany) [26] and Lodestar DX (Llusern Scientific, Cardiff, UK) [27] are leading POC platforms. Sysmex PA-100 AST POCT is a commercialized device which integrates nanofluidics and phase-contrast microscopy to detect bacteriuria (≥5×10^4^ CFU/mL) within 15 minutes and perform phenotypic AST within 30–45 minutes [28]. It monitors bacterial growth in microchambers with antibiotics, analyzing responses according to EUCAST breakpoints. Optimized for *E. coli*, *K. pneumoniae*, *P. mirabilis*, *E. faecalis*, and *S. saprophyticus*, it provides true phenotypic assessment and classifies results as susceptible (S), I (susceptible, increased exposure), R(resistant), or not applicable (NA)m. It uses a proprietary classification algorithm to group isolates (*Enterobacterales, Staphylococcus, Enterococcus*) and apply the corresponding breakpoints. Non-target species may be detected but could yield inaccurate AST interpretations if resistance is near the breakpoint [2], [28]. In other words, Sysmex PA-100 AST POCT offers results as positive or negative response for microorganisms isolation. The bacterial are in different channels in the device and according to the inhibition by antibiotics, it is informed as susceptible or resistant. The results regarding antibiogram may help in the differentiation to fungi. There is some research investigating the concordance with urine cultures [28].

In comparison, the Lodestar DX employs loop-mediated isothermal amplification (LAMP) for DNA detection of major uropathogens within 40 minutes. Its target organisms include *E. coli*, *Enterococcus* species, *S. saprophyticus*, *P. aeruginosa*, *P. mirabilis*, and *Klebsiella*. A limitation is that it provides only genotypic data; no phenotypic AST; and there may be possible genotype–phenotype mismatch [27], [29].

fASTest^®^ microfluidic system measures single-cell growth dynamics to deliver phenotypic AST in <30 minutes with clinically relevant sensitivity (10^4^ CFU/mL), applicable to multiple body fluids. The single-cell averaging strategy minimizes readout noise and enables reliable detection in 10–30 min, even in <10 min without precultivation. fASTest^®^ extends beyond *E. coli* to other uropathogens such as* K. pneumoniae* and *S. saprophyticus*. The platform offers the potential to identify polymicrobial infections through characteristic broadening of growth rate distributions and contaminants can often be excluded by differences in morphology and growth dynamics [30]. Additional POC tests include dipstick-based tools (Dip UTI, TestCard, UriScreen™), which measure indirect markers (nitrites, leucocytes, catalase), offering 2–3 min results via smartphone readout, though lacking pathogen or AST data. In contrast, culture-based systems such as Flexicult™ and Uricult^®^Trio, DipStreak^®^, and DiaSlide^®^ provide semi-quantitative identification and AST within 16–24 h, suitable for surveillance but not rapid therapy guidance [6]. Two randomised controlled trials evaluated Flexicult Human in uncomplicated UTI in women. It demonstrated pooled sensitivity of 79% and specificity of 67%, with a reduction in initial antibiotic prescribing and high accuracy for phenotypic AST supporting its potential role in guiding targeted therapy rather than empirical treatment. Uricult^®^ Trio was reported to be easy to use and convenient, but available studies provided limited data on its impact on clinical management, particularly in cases of asymptomatic bacteriuria [31].

The Vivalytic system, currently under development by Bosch Healthcare Solutions GmbH, uses PCR-based microarrays to identify nucleic acids uropathogens from selected uropathogenic species at concentrations above 10^4^–5×10^4^ CFU/mL and resistance genes in ~146 minutes, showing >90% diagnostic accuracy for *E. coli*, *E. faecalis*, *Proteus *spp., *P. aeruginosa*, and *K. pneumoniae* (81–88% for resistance markers) [26]. Limitations include reduced performance after transport, false negatives at low bacterial loads, and a 9–11% invalid rate. 

Recent studies also demonstrated ultra-rapid AST directly from positive *E. coli* blood cultures, providing ciprofloxacin and gentamicin susceptibility results within 30 minutes, substantially outperforming conventional methods. Baltekin et al. [32] reported concordance rates of 92% for ciprofloxacin and 84% for gentamicin (n=59), with valid results in 86% of cases. When tested on clinical isolates, 30-minute concordance reached 86% and 96%, respectively, increasing to 92% and 100% at 60 minutes. These findings confirm that reliable susceptibility results can be obtained as early as 20 minutes using time-dependent thresholds, allowing near real-time reporting and markedly outperforming conventional 4-hour AST assays in clinical laboratories.

## Discussion

UTIs remain among the most frequent bacterial diseases, with *E. coli* as the predominant etiological agent. Conventional diagnostic methods, although accurate, require 48–72 hours for results and depend on centralized laboratories, specialized personnel, and strict sample handling. These limitations promote empirical antibiotic use, which often leads to inappropriate therapy and fuels AMR [33].

Within this context, POC diagnostics have emerged as valuable alternatives, allowing rapid, on-site analysis without the need for complex laboratory infrastructure. POC tools substantially shorten turnaround time, facilitating early, evidence-based decisions and strengthening antibiotic stewardship efforts [6].

Among these technologies, the Sysmex PA-100 system represents one of the most clinically validated advances. Combining nanofluidics and phase-contrast microscopy, it detects bacteriuria within 15 minutes and performs phenotypic AST within an additional 15–30 minutes. It demonstrated enhanced clinical performance in economic analysis, reducing first-line antibiotic failure rates due to its high concordance with standard microbiological methods [28]. Multicenter evaluations demonstrated high concordance with conventional microbiology. A Spanish observational study validated its high sensitivity and specificity, reporting optimized treatment decisions in 78.4% of cases with PA-100, compared to 58.3% under standard clinical practice (p<0.0001). The device markedly reduced unnecessary antibiotic prescriptions: among culture-negative samples, it recommended no treatment in 99.3% of cases versus 49.6% by clinicians (p<0.0001), even among culture-positive patients, the device suggested non-treatment in 34.3% of cases versus 15.4% by clinicians (p=0.0002), and reduced inappropriate antibiotic selection from 18.2% to 2.1% (p<0.0001) [28]. Another study in women with uncomplicated UTI showed a sensitivity of 84.0% (65.7% when operator-dependent) and specificity near 99% [2]. Overall categorical agreement for AST ranged from 85.4% (95% CI: 75.9–92.2%) for ciprofloxacin to 96.4% (CI: 89.9–99.3%) for trimethoprim [28]. 

From a health economic perspective, the NICE-commissioned systematic review of 2023 found that evidence to date was insufficient to determine the cost-effectiveness of POCTs for UTI in primary care [34]. However, Medina-Polo et al. in 2024, demonstrated that full implementation of Sysmex PA-100 in Spain yielded substantial cost benefits. A budget impact model estimated annual savings of € 323.7 million (€ 119/patient) including AMR-related costs and € 971 million over three years. Even when excluding AMR, projected savings remained significant at € 4.25 million per year (€ 1.57/patient) [2]. Sensitivity analyses confirmed the robustness of these findings, underlining the system’s potential not only to optimize patient-level outcomes but also to relieve economic burdens associated with UTI mismanagement and AMR propagation. 

However, there are limitations. The device performance may decrease outside the validated sample specifications and it reports bacteriuria only above 50,000 CFU/mL, potentially missing lower yet clinically relevant counts. Its classification is restricted to broad groups (*Enterobacterales, Staphylococcus, Enterococcus*) without full species identification, and applies uniform breakpoints across Enterobacterales, which may misclassify atypical strains. Performance is slightly reduced in Gram-positive isolates such as *S. saprophyticus* and *E. faecalis *[35]. These methods are mainly suitable for uncomplicated UTIs because the most advanced and clinically validated commercial phenotypic platforms analysed in this review such as Sysmex PA-100 System are optimized for a highly specific panel of common uropathoges (*E. coli, K. pneumoniae, P. mirabilis, E. faecalis*…). These systems carry a high risk of missing low yet highly relevant pathogen counts in complicated cohorts so standard centralized cultures are mandatory for complicated UTUs until broader panels and lower analytical thresholds are clinically validated. 

Continuous refinement and validation across diverse patient populations are warranted.

When compared with earlier POC platforms, Sysmex PA-100 offers superior diagnostic and clinical robustness. Paper-based devices are highly portable, inexpensive, and can reach PCR-level sensitivities (as low as 10 CFU/mL), but their accuracy depends on detection chemistry and often requires several hours of enrichment. In contrast, PA-100 consistently identifies bacteriuria ≥5×10^4^ CFU/mL within 15 minutes and provides phenotypic AST in 30 minutes. Similarly, dipstick and smartphone-based assays (e.g., Dip UTI, TestCard, UriScreen™) enable rapid home screening in <3 minutes and support early triage, but lack pathogen identification and AST capability, making them unsuitable for therapeutic guidance in confirmed or complicated cases. These tests may serve as preliminary screening tools, while the Sysmex PA-100 provides the necessary depth of information for precise therapeutic interventions. 

LFAs, such as RapidBac^®^, further illustrate the trade-off between accessibility and diagnostic depth. RapidBac^®^ achieves >95% sensitivity for Gram-negative bacteria and 94% specificity within 20 minutes, comparable in speed to Sysmex PA-100. Nevertheless, LFAs are limited to pathogen detection and cannot provide phenotypic AST [14]. Sysmex PA-100, by directly observing bacterial growth in antibiotic microenvironments, provides both detection and phenotypic AST, being a decisive advantage for managing complicated or recurrent UTIs, where resistance patterns critically influence outcomes [28]. The cut-off point was defined in 50.000 CFU/ml, based on accepted positive cut-off for urine infections 100,000 CFU/mL. It is true that lower level of bacteria can be found. However, Tarrés et al. [28] reported a sensitivity and specificity for detection of microbiologically confirmed bacteriuria were 84.0% (89/106; 95% CI: 75.6–90.4%) and 99.4% (155/156; 95% CI: 96.5–100%), respectively, for bacterial species within the analyser specifications. 

Emerging microfluidic and hydrogel-based POC systems, including stack-pad assays, chitosan hydrogels, and electrochemical impedance spectroscopy (EIS) systems, offer remarkable analytical performance, with detection limits as low as 10^2^ CFU/mL and identification within five minutes – performance that far exceeds the analytical sensitivity of Sysmex PA-100. The fASTest^®^ platform, based on single-cell microfluidic imaging, delivers AST in <30 minutes at clinically relevant thresholds (10^4^ CFU/mL). It can also assess polymicrobial samples and various body fluids, including blood and cerebrospinal fluid [30]. 

Sysmex and fASTest direct susceptibility testing from urine samples may be affected by non-standardized inocula and occasional polymicrobial infections. However, both options determine susceptibility based on the detection of bacterial growth inhibition in the presence of antibiotics, reflecting the overall response of the microorganisms present in the sample. Despite these limitations, Tarrés et al. [28] reported a high agreement with conventional urine culture results (85.4–96.4%).

Despite excellent laboratory performance, these devices remain at proof-of-concept stages without regulatory approval or clinical integration comparable to Sysmex. EIS-based biosensors, validated for both Gram-negative (*E. coli*) and Gram-positive (*S. aureus*, including MRSA) pathogens, achieve susceptibility determination within 2–2.5 hours – substantially faster than culture – but remain limited to pilot testing [36], [37].

Molecular systems such as Lodestar DX and Bosch Vivalytic rely on loop-mediated isothermal amplification (LAMP) and PCR microarrays, respectively, detecting uropathogens and selected resistance genes within 40–146 minutes, respectively. While these provide high analytical specificity, their focus on predefined genetic targets can overlook emerging resistance mechanisms or expression-level variations [26], [27]. PCR-based methods indicate genotypic susceptibility by detecting specific resistance genes or mutations. While they are highly accurate for confirming resistance, they cannot definitively prove susceptibility; missing a resistance gene does not guarantee the bacteria will respond to treatment in a clinical setting. For analysis of antibiotic susceptibility, urine culture remains as the gold standard [38]. Sysmex overcomes this constraint by assessing live bacterial response to antibiotics, yielding a true phenotypic profile within an actionable timeframe. The Vivalytic UTI POCT, though still under evaluation, integrates all processing steps into a disposable cartridge, eliminating the need for laboratory referral and demonstrating >90% accuracy for major uropathogens and 81–88% for resistance gene detection [26].

Other commercially available, home-use, culture-based POC tests (Flexicult™, Uricult^®^ Trio, and DipStreak^®^) offer advantages such as affordability, pathogen identification, resistance profiling, and quantification. Although slower, these remain cost-effective and adaptable for resource-limited settings. Similarly, ongoing adaptations of multiplex PCR platforms such as Roche SeptiFast^®^, bioMérieux FilmArray^®^, and Cepheid GeneXpert^®^ show potential for urinary diagnostics, yet they currently lack bacterial quantification, a parameter essential for clinical decisions [6]. Culture-based POCTs demonstrate better diagnostic accuracy than dipstick-based tools, although robust evidence of clinical benefit is still needed to clarify their real impact on antibiotic stewardship, patient outcomes, and cost-effectiveness, particularly in primary care.

Overall, while emerging technologies exhibit impressive analytical capabilities, most lack extensive clinical validation and regulatory approval. Sysmex PA-100 remains the only CE-marked phenotypic POC system optimized for urinary pathogens, integrating EUCAST-based interpretive algorithms that ensure standardized and reproducible reporting. Its dual capacity for rapid bacteriuria detection and AST supports rational antibiotic use and has demonstrated both clinical effectiveness and economic efficiency. Broader adoption of such validated POC systems could play a pivotal role in combating AMR by reducing diagnostic delays and minimizing empirical prescribing [2], [28]. 

Among rapid POCTs, Lodestar DX demonstrated promising sensitivity and specificity for the detection of *E. coli* in a laboratory setting, whereas Uriscreen showed only modest accuracy and UTRiPLEX exhibited poor sensitivity despite high specificity. However, the methodological quality of these studies was limited, with high risk of bias, and head-to-head comparisons were rare, meaning that relative test performance is uncertain [31].

In this line, future evaluations such as the ongoing TOUCAN prospective study in UK primary care, are expected to provide real-world evidence regarding diagnostic accuracy and usability of next-generation POC systems, including Sysmex PA-100 and Lodestar DX. Although focused on diagnostic performance rather than patient outcomes, these studies will help clarify the role of rapid phenotypic testing in improving treatment decisions for female uncomplicated UTI [29].

In summary, the Sysmex PA-100 system exemplifies the optimal balance between analytical sophistication and clinical practicality. While experimental microfluidic and molecular platforms may surpass it in sensitivity or breadth of detection, Sysmex uniquely delivers clinically actionable, phenotypic AST within 45 minutes using a fully automated workflow. This represents a significant advancement in POC UTI management, offering a practical, validated, and cost-effective solution that enhances antimicrobial stewardship, reduces inappropriate antibiotic use, and improves patient outcomes.

## Conclusion

POC diagnostic technologies represent a transformative advance in the rapid detection and management of UTIs supporting antimicrobial stewardship. Despite low adoption rates globally, POC systems like PA-100 are shown to be cost-effective and resource-efficient, and its broader implementation is strongly recommended for uncomplicated UTIs diagnosis. Future research should prioritize well-designed randomized controlled trials integrating diagnostic accuracy, clinical outcomes, antimicrobial resistance metrics, and economic endpoints. Such evidence will be essential to define the true clinical value of POCTs within urological and primary care practice. Such developments will be critical to maximizing their impact in both inpatient and outpatient settings, ultimately contributing to reduced AMR prevalence and improved patient outcomes. 

## Notes

### Authors’ ORCIDs

- Silvia Juste-Alvarez: 0009-0002-9591-2610
- José Medina-Polo: 0000-0003-3626-8669

### Ethics statement

This systematic review was conducted in accordance with the PRISMA guidelines. As no human or animal subjects were involved, ethical approval and informed consent were not applicable.

### Competing interests

The authors declare that they have no competing interests.

## Figures and Tables

**Table 1:**
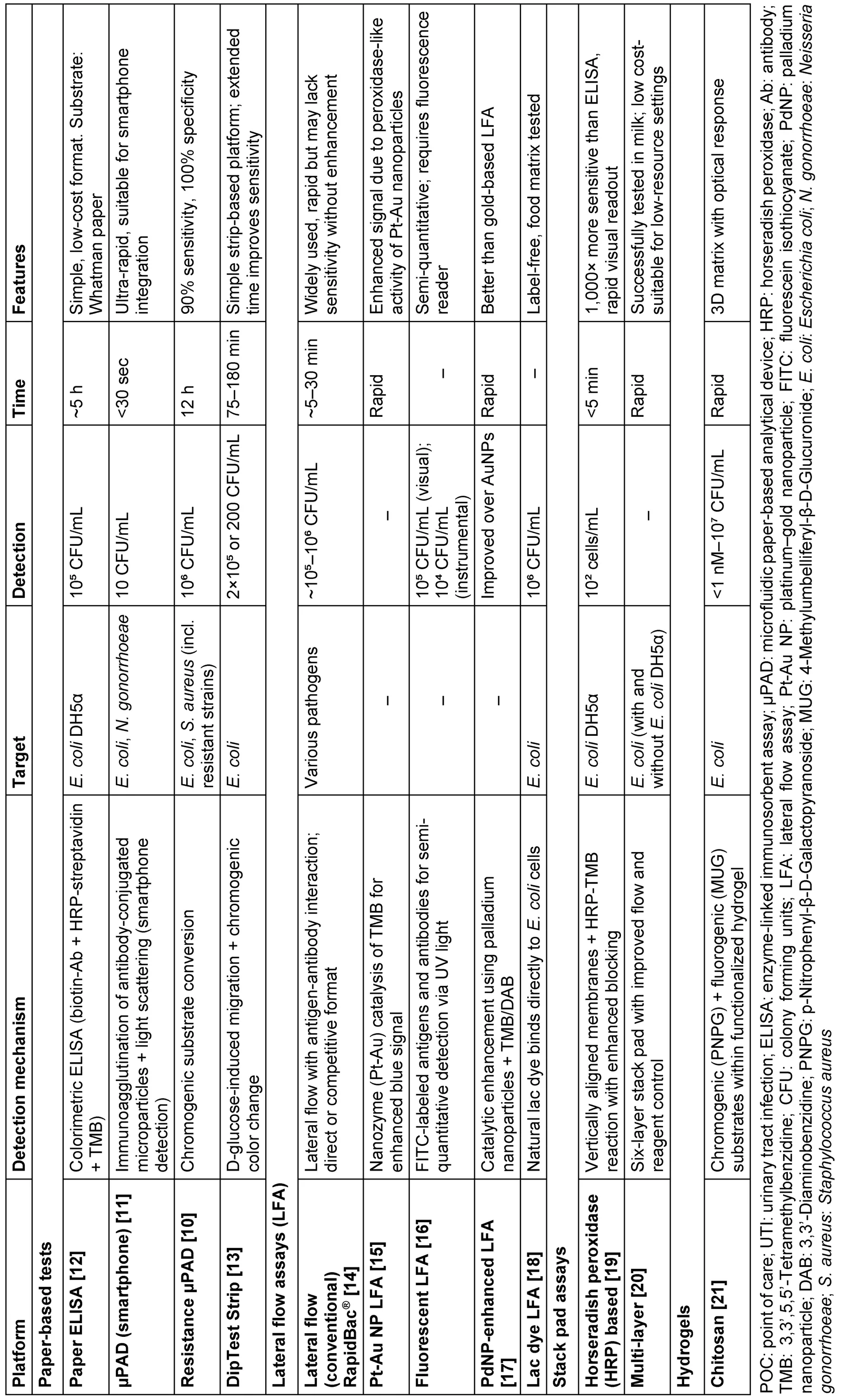
POC diagnostic tools for UTI pathogen detection
